# Collaborative Governance for Innovative Environmental Solutions: Qualitative Comparative Analysis of Cases from Around the World

**DOI:** 10.1007/s00267-022-01642-7

**Published:** 2022-05-23

**Authors:** Emma Avoyan

**Affiliations:** Radboud University, Nijmegen, Netherlands

**Keywords:** Collaborative governance, Innovation, Environmental management, Qualitative comparative analysis (QCA)

## Abstract

It is common understanding that to address pressing environmental issues and ensure sustainable environmental management innovative solutions are required. Many studies have striven to understand which governance conditions enable generation of innovative solutions. However, there are very few studies in the field of management and public administration studies that investigate the combined, interactive effects of a suit of conditions on the likelihood of innovative solutions. This article uses the method of qualitative comparative analysis (QCA) to investigate the complex causality of collaborative governance and innovative solutions. More specifically, it examines the combination of conditions of collaborative process, leadership, institutional design and knowledge sharing, and their joint effects on the presence or absence of innovative solutions. An analysis of 16 cases of environmental endeavors with a goal of generating innovative solutions and extracted from Collaborative Governance Case Database shows that there are 3 possible configurations or paths leading to innovative solutions. Various combinations of the above-mentioned conditions can in fact be sufficient for generating innovative solutions. The configurations provide insight into which collaborative conditions deserve attention when aiming for innovation in the field of sustainable environmental management.

## Introduction

It is widely recognised nowadays that human activities are putting increasing pressures on our living environment (OECD 2012). We need to be aware and become more responsible with environmental management having no alternative other than conforming with the principles of sustainability. It is also recognised that this can be done only by creating new ideas and innovations (Maier et al. 2020), a challenge that requires more than technological innovation. Although technological innovations are considered pivotal to realizing sustainability transitions (Paredis 2011), major changes are needed also in the governance processes, multi-level structures and institutions, and the behaviour of actors involved in developing innovative solutions for sustainable environmental management (Ansell and Torfing 2014). For that reason, governments, environmental managers, private organisation and various interest groups are searching for novel solutions (e.g. innovative measures, systems, structures, processes) to address local to global-scale environmental changes, such as climate change, widespread pollution or resource extraction as well as the impacts these pressures are creating for systems in place to meet fundamental societal needs (Frantzeskaki and Loorbach 2010; Westley et al. 2011).

However, most environmental problems being wicked (e.g. climate change), are particularly challenging for the governmental officials and other actors to address (Rittel and Webber 1973). They are generally imprecise and result in problem shifting when a solution to one problem creates new problems elsewhere (Rakhyun and van Asselt 2016). Addressing such environmental problems through conventional solutions is rather implausible and calls for new, innovative solutions (Ansell and Torfing 2014; Crosby, ‘t Hart, and Torfing 2017). Generating and implementing innovative solutions on the other hand requires not only financial resources, but also collective agreements, expertise, knowledge, procedural arrangements and many other capacities which collaborative governance can help to acquire (Steinacker 2009; Torfing et al. 2020). Presumed benefits of collaborative governance such as establishing partnerships, sharing knowledge, accumulating resources and expertise are particularly highlighted in different studies to be critical for generating innovative solutions (e.g. Crosby et al. 2017). Furthermore, understanding the virtues and the effects of collaborative governance on environmental innovation has become a needful line of study in management research (Araújo and Franco 2021).

Yet, empirical studies establishing the positive impact of collaborative governance on the development and production of innovative solutions for environmental management are lacking. In particular, little is known about collaborative conditions as well as combinations thereof that enable or constrain development of innovative solutions. The present article addresses this gap by linking innovative solutions for environmental management, or else environmental innovations (new technologies, policies, products and practices) to collaborative governance. A fuzzy set qualitative comparative analysis (fsQCA) of 16 cases of collaborative governance is conducted to test and compare how collaborative process, leadership, institutional design and knowledge sharing, impact the development and production of innovative solutions for environmental management in the context of selected mix of cases. The data is drawn from Collaborative Governance case Databank that provides a repository for collaborative governance case studies from around the world (Douglas et al. 2020). I make no claims about the outcomes of environmental innovation in the selected cases, for example whether they improve environmental management or reduce environmental externalities. Instead, I consider developed innovative solutions (if any) within the studied cases as outputs of collaborative processes and investigate the conditions of collaborative governance that are necessary and/or sufficient for these outputs to come about.

This study offers two contributions to the literature of collaborative governance, innovation and environmental management. First, the findings demonstrate the important role of institutional design of collaborative governance in the efforts to generate innovative solutions. Almost all successful cases that have generated innovative solutions acquire functional institutional designs. Second, the findings demonstrate that sole presence of institutional design (or the other conditions) is neither necessary nor sufficient for it. Only a combination of institutional design with knowledge sharing of collaborating parties or productive collaborative process and leadership results in innovation solutions in the studied cases. This finding suggests there might be different pathways to generating innovative solutions and highlights the interconnectedness of collaborative conditions. This article begins with conceptualizing innovative solutions followed with a section that links innovative solutions with collaborative governance and the select group of conditions. Then I present the data used for the article and the method of Qualitative comparative analysis (QCA) that was applied to analyze the data. This section is then followed by a presentation of findings and conclusions.

## Conceptualising Innovative Solutions

There are diverse conceptions of innovation. It is a complex phenomenon being studied by different academic disciplines, including economics, engineering and sociology. Generally, innovation is defined as the development and implementation of new solutions that break with the dominant ideas and practices in a particular context (Hartley, Sørensen, and Torfing 2013). At the organisational level, scholars have approached the construct of innovation as both the development and implementation of novel ideas and/or behaviours related to products, services, systems or practices (e.g. Damanpour and Schneider 2006; Hartley et al. 2013). In this respect, the generation, adoption and implementation of innovation are understood as processes or outputs that are meant to result in outcomes the innovative solutions are developed for (Walker 2008). In other words, innovation is not a goal per se, but a way or an instrument to reach the goal of creating different public values (Torfing et al. 2020). One of the dimensions of public values, the environmental dimension, is the focus of this article.

An increasing number of scholars are studying innovation for sustainable environmental management from various perspectives (Clausen and Fichter 2019; Fernando et al., (2019); Paredis 2011; Wagner 2007; Weber and Hemmelskamp 2005). Labelling innovative solutions differently (e.g. environmental innovations, eco-innovations, green innovations, ecological innovations), they all highlight the clear environmental aim of the solutions (for overview: Araújo and Franco 2021). Unlike other issues, such as economic development, which are receptive to trade-offs, responses to environmental issues often require a different approach to planning and decision making (Coffey 2021). This is evident in debates about the need for integrated rather than sectoral responses, and transformative change, which represent considerable challenge given the contested nature of environmental politics (Díaz et al. 2019). Environmental innovations have similarities and differences in relation to other types of innovation (e.g. new technology) generated by organisations (Araújo and Franco 2021). One considerable difference lies in the knowledge-sharing and collaboration practised for environmental innovation, which differ greatly from conventional innovation (González-Moreno et al. 2019). For this article, I approach innovative solutions as outputs of collaborative processes generated with technologically new or modified products and processes or novel solutions and/or improvements in existing policies, programs and practices for sustainable and optimal environmental management.

## Collaborative Governance as Determinant for Innovative Solutions

The forces moving innovation for environmental management are of great importance for politicians, managers, businesses and society, and therefore, the literature contains important research investigating the determinants and governance modes of generating innovative solutions. There are abundant reports about political and business leaders, public entrepreneurs and private contractors or else individual innovators being critical determinants for innovative solutions (e.g. Doig and Hargrove 1990; Roberts et al. 1996). Yet, without downplaying the important role of single innovators, more recent research argues that innovative solutions are frequently an output of partnerships between a range of public and private actors and collaborative governance (Meijer 2014; Torfing et al. 2020) understood as “a collective decision-making process based on more or less institutionalized interactions between two or more actors that aims to establish common ground for joint problem solving and value creation” (Douglas et al. 2020, p. 498). Multi-actor and multi-sector collaborative governance that attempts to solve an environmental problem or issue in a consensual way often can foster innovation (Ansell and Torfing 2014; Hartley et al. 2013). The idea that multi-actor collaboration can spur innovation is well supported by innovation system theory (Hekkert et al. 2007) as well as theories of public innovation (Gieske et al., (2016)). Due to the high cost and also the high risk of environmental innovation, organisations are reluctant to embark on its activities in practice (Weber and Hemmelskamp 2005). The high cost and risk can be lessened by important choices throughout the process, such as deciding to undertake the development and implementation of environmental innovation in collaboration with other organisations. In that effort towards ensuring sustainable environmental management, public and private organisations more frequently engage with each other and increasingly with the society, instead of relying on traditional cooperating or coordinating mechanisms.

There are also many other claims in favor of collaborative innovation including the argument that multi-actor collaboration facilitates the production of a more exact interpretation of the issue at hand, stimulation of mutual learning, creation of shared ownership over innovative solutions, coordinated implementation etc. (Torfing 2016). Furthermore, resources accumulated or brought to the process by different collaborating parties may be consequential. In a more traditional cooperative or coordinative settings, resources of parties (e.g. knowledge and information) may only be exchanged to achieve individual goals, while in collaborating settings the resources and assets are pooled in support of collective goals (McNamara 2012). These resources may include financial, technical, scientific or logistical assistance and organizational support, and knowledge and skills. They may be combined or intentionally distributed among the participating organisations to generate desired interventions and outputs (Berardo 2014) such as innovative solutions. Drivers of innovation manifest both from single sources and from combinations of them, working collaboratively, or iteratively, to generate innovation between collaborating parties (Beynon et al. 2016). Furthermore, the collaborative process tends to disturb the established practices, a condition for innovation, according to Torfing et al. (2020). The authors also argue that communication, institutional design and good leadership are important conditions for the development of innovation. For this study, I have selected four collaborative conditions that I expect to make a positive contribution to the development of innovative solutions.

### Collaborative Process

Collaborative engagement processes illustrate behavioral interactions among collaborating parties. These processes are at the core of jointly generating innovative solutions as they provide a venue where actors position themselves toward the others, build trust, internal legitimacy as well as commitment to the idea that brought them together (Ansell and Gash 2008; Emerson and Nabatchi 2015), such as innovating for enhanced environmental management. Researchers have accentuated various aspects of processes within collaborations fostering effective collaboration (for overview: Bryson et al. (2015)). Here I focus on at least three: (1) identification and/or examination of information relevant to the collaboration via regular meetings, (2) interpretation of shared meanings around that information, (3) deliberation and joint problem-solving of the addressed issues (Emerson et al. 2012). In other words, collaborative process shapes the substance of innovative solutions. I expect a productive collaborative process to contribute to development of innovative environmental solutions if it regularly offers participants opportunities to identify, deliberate and make agreements over objectives and shared meanings about innovative solutions.

### Institutional Design

If collaborative process shapes the substance, institutional design provides a structure for co-development of innovative solutions. Institutional design establishes the basic rules arrangements under which collaboration is taking place (Ansell and Gash 2008). For example, rules and procedures for co-creation and co-development is fundamental design issue as generating joint outputs and outcomes is ultimately the purpose of collaboration. Alexander conceptualize institutional design as “the devising and realization of rules, procedures, and organizational structures that will enable and constrain behavior and action so as to accord with held values, achieve desired objectives, or execute given tasks” (Alexander 2005, p. 213). In the contexts when innovation is an objective or one of the objectives of collaboration, some studies show that structural constituents of collaboratives such as rules of inclusion of divers actors may spur innovation for example in the field of urban planning (Dente et al. 2005). Practically speaking, institutional design entails the development of a regulatory framework for collaborative process, and the formation of procedural arrangement for making joint decisions and their implementation (Torfing et al. 2020). It is considered functional if the basic procedural and institutional rules/arrangements critical for the procedural legitimacy of the collaborative process are followed (Ansell and Gash 2008) and the mechanisms of co-development and implementation of joint outputs (e.g. innovative solutions) are used.

### Collaborative Leadership

The literature on collaborative governance highlights the importance of leadership in initiating and sustaining collaborative arrangements (Ansell and Gash 2008; Bryson et al. 2006; Emerson et al. 2012) and stimulating transformative learning and creative problem solving (Crosby et al. 2017; Sørensen and Torfing 2011). Public leaders may play different roles in processes of generating innovative solutions (Ansell and Torfing 2014). Sullivan and Williams (2012) argue that along with ordinary management tasks, leaders in collaborative arrangements are responsible for handling conflicts or risks to build productive relationships between parties. Hofstad et al. (2021) advance co-creation leadership form that aspires to generate collaborative innovation through undertaking different tasks (e.g. mobilizing actors who possess necessary assets for producing public value, managing risks associated with innovation, engaging in boundary-spanning activities etc.). Performing such responsibilities makes a range of leadership qualities desirable and necessary. For example, in network management literature researchers highlight a number of leadership qualities to boost collaboration, such as the ability to bring actors together and focus on enabling interactions and relationships (Edelenbos et al. 2013). Studies also highlight the role of leaders, particularly their connective capacity, in good performance of collaborative arrangements (Avoyan et al. forthcoming). Productive and performing collaborations may also rely on multiple leadership roles, rather than relying on one leader (Ansell and Gash 2008; Meijerink and Stiller 2013). In this paper, I consider the collaborative leadership being effective if it displays a management style of engaging and connecting actors and expect such leadership contributes to development of innovative solutions.

### Knowledge Sharing

Both collaborative and network governance literature also accentuate the importance of knowledge sharing (or knowledge building as a resource) to collective outputs, such as innovative solutions. Referring to knowledge, Thomson, J (2006) mention that the possibility to share knowledge among collaborating parties can itself be seen as one of the benefits of collaboration. The different kinds of knowledge (e.g. expert, local, lay) of various actors may be combined or intentionally distributed among the participating organisations to generate desired interventions and outputs (Berardo 2014) or to establish a common ground for joint learning (Gerlak and Heikkila 2011). Moreover, specific knowledge sharing and building activities that facilitate deliberation and discussion can also facilitate better understanding of novel concepts or technical measures by overcoming contested or uncertain knowledge (Emerson et al. 2012). In the context of inclusive collaborative settings for environmental management, the knowledge brought to the table by advocates of environmental interests can increase the environmental quality of outputs (Brody 2003; Newig et al. 2018), such as innovative environmental solutions. With more collaborating actors, it is more likely to accumulate different kinds of knowledge and positively influence innovative performance of collaboratives (Knudsen 2007). Furthermore, by enabling knowledge sharing, the authorities or decision making actors may get better understanding of beneficiaries and their practices, local norms or competing stakes, which will help them to first anticipate the acceptability of proposed measures and second adjust the measures to enhance the acceptability and implementability (Newig et al. 2018). In this study, I anticipate that sharing and managing the knowledge of collaborating parties contributes to the development of innovative solutions.

In sum, it is expected that all four conditions make a positive contribution to development of innovative solutions. Previous studies have tested the independent impact of different conditions on collaborative outcomes (Turrini et al. 2010; Ulibarri 2015), but we need to study the effects of competing configurations of conditions in order to understand how the presence and/or absence of different conditions combine to produce successful results, such as innovative solutions. I am interested, therefore, in the extent to which above four conditions substantiate and complement each other, and hence show signs of partial and conditional necessity. To understand the combinations of collaborative conditions that led to innovative solutions (or their absence) in the selected cases, I have applied a qualitative comparative analysis (QCA), which is detailed below following the presentation of data used for this study.

## Data and Method

### Collaborative Governance Data Bank

The data used in this study originate from the Collaborative Governance Data Bank (Douglas et al. 2020). All cases included in the data bank are examples of collaborative governance drawn from different countries and different policy domains. The data of cases, which includes both survey format with a scale from 1 to 5 and texts, is provided by different researchers studying collaborative governance. For the purposes of this article, I have selected the cases covering *Environment and climate* policy domain alone or in combination with other sectors. This strategy allowed to compile 20 cases. Furthermore, I filtered the cases based on their aim: whether the cases featured innovation being an objective/ambition. 3 cases were excluded from the analysis because the generation of innovative solutions was not the main motivation of these collaboratives. Next, I further excluded one case where data was missing. These considerations left me with 16 cases for the analysis (overview in Table 1). With this dataset, I followed Torfing et al. 2020 and chose to focus on data related to the middle of the period observed. As the main criterion for case selection was that innovation should be an objective of the collaboration, I chose those cases that had such objective in the middle of the period observed rather than start or end periods. Choosing the start point could be unreliable as collaborations may not have clearly stated goal of innovating from the start of collaborative processes, but may cultivate it after sometime when joint opportunities become clearer. Likewise, selecting the end of period may not be correct, as this period may coincide with a phase of collaboration when participants switch to other objectives, because possibly they already generated innovative solutions among other reasons (Torfing et al. 2020). Subsequently, the middle of period of scores was also used for the conditions.

**Table 1:** Overview of cases

Case label	Name	Type of data collection	Jurisdictional level	Country /region	Policy domain in addition to environment & climate
Case01	2010 & 2011 Ash Cloud Crises	Documents	National, International, Supranational (UN, EU, etc.) Multi-level	Europe	Security & Public Safety, Technology & Transport
Case02	Public-private-people collaboration in peri-urban area development	Documents, Interviews, Observations Social	Local, Regional	Netherlands	Culture/Leisure, Education, Infrastructure & Planning
Case03	Independent Inquiry into Container Deposit Legislation in NSW	Documents, Interviews, Observations Social	Regional	Australasia	–
Case04	Desert Tortoise Habitat Conservation Planning	Documents, Interviews	Local, Regional, National	North America	–
Case05	Collaborative governance in Vietnam flooding	Documents, Interviews, Observations Social, Surveys	Local, Regional, National	East Asia, Vietnam	–
Case06	Chinchina Besin Management Plan	Documents, Interviews	Regional	Colombia	–
Case07	Blackfoot Challenge	Documents, Interviews, Observations Social,	Local, Multi-level	North America, USA	Agriculture Culture/Leisure, Economy & Trade
Case08	Area C - Milan	Documents, Interviews	Local	Italy	Infrastructure & Planning, Technology & Transport
Case09	Baker River Hydroelectric Project	Documents, Interviews, Network data, Surveys	Local, Regional, multi-level	North America, USA	Infrastructure & Planning
Case10	Lake Tahoe	Documents, Interviews	Local, Regional	North America, USA	–
Case11	Tampa Bay	Documents, Interviews	Local, Regional	North America, USA	–
Case12	Tillamook Bay, Oregon	Documents, Interviews	Local	North America, USA	–
Case13	Narragansett Bay (RI)	Documents, Interviews, Observations Social	Regional	North America, USA	–
Case14	Delaware Inland Bays	Documents, Interviews, Observations Social	Local	North America, USA	–
Case15	Rhode Island’s Salt Ponds	Documents, Interviews, Observations Social	Local, Regional	North America, USA	–
Case16	Grebbedijk	Documents, interviews, surveys	Local	Netherlands	Infrastructure & Planning

The detailed description of cases can be accessed at https://collaborativegovernancecasedatabase.sites.uu.nl/

### Qualitative Comparative Analysis

Given the aim of this study and its comparative nature, I favoured a method that allows to extract different configurations of collaborative conditions enabling innovative solutions in the studied cases. Qualitative comparative analysis (QCA) is a set-theoretic method of systematically comparing multiple cases in terms of their membership (or non-membership) in the sets of conditions and the outcome of interest (Ragin 2008; Rihoux and Lobe 2012). In QCA terminology, the terms “condition” and “outcome” are in principal equivalent to “independent variable” and “dependent variable” terms used in other research contexts, such as statistical methods (Schneider and Wagemann 2012). However, unlike conventional statistical analysis of investigating the independent effect of a variable on the outcome of interest, QCA allows for the use of both in-depth qualitative case knowledge and quantitative data to identify varying configurations of conditions and their joint effect on the likelihood of an outcome (Ragin 2012; Schneider and Wagemann 2012). The ambition of QCA is to distinguish necessary (whenever the outcome is present, the condition is also present) and sufficient (whenever the condition or configuration of conditions is present, the outcome is also present) conditions that justify the outcome of interest (Schneider and Wagemann 2012).

QCA provides a choice between its two main variants: crisp (used to deal with binary data and indicates either present/1 or absent/0 set membership) and fuzzy analysis (used to capture the complexity in cases by level or degree and thus indicates also the values within the crisp range of 0–1) (Schneider and Wagemann 2012). I have performed a fuzzy-set variant of qualitative comparative analysis (fsQCA) in fsQCA 3.1b software package (Ragin and Davey 2019). FsQCA is preferred over crisp set QCA as it allows for different degrees of membership in sets rather than only using dichotomous sets as with crisp analysis. FsQCA includes more nuanced information distinguishing between differences in cases both in kind and in degree. This results in a higher content validity (Schneider and Wagemann 2012). Moreover, fsQCA identifies not just necessary and sufficient conditions, but also so-called INUS conditions that are insufficient on their own but are necessary parts of solutions and can explain the outcome (Schneider and Wagemann 2012). This phenomenon, known as “equifinality,” is difficult to capture in statistical analysis. For instance, one configuration for a given outcome may require the presence of a condition, while a second configuration for the same outcome may require the absence of the same condition. FsQCA is designed to allow and identify equifinal solutions (Fainshmidt et al. 2020). Finally, fsQCA allows for differentiating between core and peripheral conditions as well as “do not care” situations (Fiss 2011). Core conditions indicate a strong causal relationship with the outcome while peripheral conditions display a weaker relationship. The “do not care” situation indicates that the condition may either be present or absent and it does not play a role in a specific configuration. This distinction of causal conditions enables a fine-grained examination of causal processes determining what really matters and to what degree (Fiss 2011).

### Operationalization: Calibration of the Outcome and Conditions

Causal conditions and outcomes in fsQCA assume set membership values from 0 (“fully absent”) to 1 (“fully present”). To achieve this, fsQCA requires a process called “calibration”, entailing a refinement of the operationalization and setting of the set membership thresholds (Ragin 2008). Therefore, I first created the data matrix (Table 7) based on the scores of the indicators (survey questions) for the outcome and the conditions (Table 2). Then I calibrated the outcome and conditions specifying three qualitative anchors: the threshold for full membership (1), the threshold for full non-membership (0) and a crossover point (0.5) for each of the conditions included in the analysis (Ragin 2008). Membership scores close to 1 (e.g. 0.9) indicate strong but not full membership, while membership scores below 0.5 but greater than 0 (e.g. 0.3) indicate that the condition is still weak member of the set. The crossover point 0.5 is the point of maximum ambiguity. The Collaborative Governance Data Bank uses a Likert scale to score the indicators of different conditions/variables of collaborative governance. I set the endpoints of the 5-point Likert scales as the two qualitative anchors for calibration of full membership (value 5) and full non-membership (value 1). The crossover point was then calculated by observing the median score of each indicator. The literature indicates that sample-based calibration should be avoided whenever possible (Greckhamer et al. 2018). However, in the case of survey-based data coming from individuals’ self-reported perceptions, this choice can be justified as a “median” cross-point, which is better than simply using the midpoint of the scale. Table 2 includes a brief explanation of my choices for calibration, while Table 3 displays the data matrix after performing the calibration procedure.

**Table 2:** Overview of operationalization and calibration of outcome and conditions

Conditions	Description	Associated survey questions (Likert scale from 1 to 5)	Calibration	Explanation of calibration
Collaborative process (label in QCA: PRCS)	Process that regularly offers participants opportunities to identify, deliberate and make agreements over objectives and shared meanings about innovative environmental solutions.	Q.38. To what extent did the participants engage in face-to-face dialogue through holding regular meetings with good attendance? (1 = Very infrequently, 5 = Very frequently). Q.40. To what extent did the participants in the collaborative process invest in joint fact finding? (1 = Very little investment, 5 = Very high investment). Q.42. To what extent did the collaborative process focus on the alignment of interests and values among all actors? (1 = Very weak focus, 5 = Very strong focus). Q.43. To what extent did the collaborative process focus on joint problem-solving (e.g. joint problem framing, co-designing solutions)? (1 = Very weak focus, 5 = Very strong focus).	Full non-membership (0) = 1 = limited or unproductive collaborative process Crossover point (0.5) = 3.6 Full membership. (1) = 5 = productive collaborative process.	For the calibrated score of collaborative process a four-item (4 survey questions) composite measure obtained as the average score of four questions was used. The crossover point was calculated on the basis of the median of the scores.
Collaborative leadership (LEAD)	A leader that displays a management style of engaging and connecting actors.	Q.32. To what extent was the leadership effective in convening / bringing together the relevant and affected actors? (1 = Highly ineffective, 5 = Highly effective) Q. 35. To what extent was the leadership effective in creating and realizing concrete opportunities for creative problem resolving? (1 = Highly ineffective, 5 = Highly effective).	Full non-membership (0) = 1 = ineffective collaborative leadership Crossover point (0.5) = 4 Full membership (1) = 5 = effective collaborative leadership.	For the calibrated score of collaborative leadership a two-item (2 survey questions) composite measure obtained as the average score of two questions was used. The crossover point was calculated on the basis of the median of the scores.
Institutional design (DSGN)	Procedural and institutional rules/arrangements that enable co-development of innovative solutions.	Q.25. To what extent were the procedural ground rules observed and applied? (1 = Very rarely applied, 5 = Almost always applied ground rules). Q.56. To what extent collaboration use any of the following forms of collaborative governance: - co-develop: jointly create and arrange policies, services, or regulations (1 = Very low extent, 5 = Very high extent).	Full non-membership (0) = 1 = nonfunctional institutional design Crossover point (0.5) = 3.75 Full membership (1) = 5-functional institutional design.	For the calibrated score of institutional design’s functionality a two-item (2 survey questions) composite measure obtained as the average score of two questions was used. The crossover point was calculated on the basis of the median of the scores.
Knowledge sharing (KNWL)	Sharing and managing knowledge for developing innovative solutions.	Q.41. To what extent did the participants in the collaborative process invest in knowledge sharing? (1 = Very infrequently, 5 = Very frequently).	Full non-membership (0) = 1 = no or limited knowledge sharing Crossover point (0.5) = 4 Full membership (1) = 5 = considerable knowledge sharing.	For the calibrated score of knowledge sharing one survey question was used with the crossover point set on the median of the score.
Outcome				
Innovative solutions (INNV)	Perceived creation of innovative solutions for environmental management.	Q.55. To what extent did the collaboration produce the following outputs or outcomes? -Create innovative solutions in existing policies, programs, practices (1 = Very low, 5 = Very high).	Full non-membership (0) = 1 = no or limited innovative solutions Crossover point (0.5) = 4 Full membership (1) = 5 = innovative solutions.	For the calibrated score of innovative solutions one survey question was used with the crossover point set on the median of the score.

**Table 3:** Calibrated data matrix

Case label	Innovative solutions	Collaborative process	Collaborative leadership	Knowledge sharing	Institutional design
Case01	0.51	0.45	0.51	0.51	0.14
Case02	0.51	0.95	0.95	0.51	0.65
Case03	0.27	0.05	0.08	0.05	0.01
Case04	0.27	0.45	0.51	0.95	0.14
Case05	0.05	0.06	0.23	0.12	0.14
Case06	0.27	0.12	0.05	0.51	0.14
Case07	0.51	0.79	0.95	0.95	0.95
Case08	0.95	0.29	0.82	0.95	0.95
Case09	0.51	0.87	0.82	0.95	0.65
Case10	0.95	0.45	0.23	0.51	0.86
Case11	0.51	0.69	0.82	0.27	0.65
Case12	0.51	0.56	0.35	0.27	0.35
Case13	0.27	0.22	0.05	0.12	0.14
Case14	0.27	0.56	0.51	0.51	0.35
Case15	0.95	0.69	0.95	0.27	0.95
Case16	0.95	0.79	0.95	0.95	0.86

## Analysis

I have first assessed the necessity of each separate condition for the occurrence of innovative solutions (Table 4). The commonly accepted consistency threshold here is 0.9 (Schneider and Wagemann 2012): if a condition has a consistency score above this threshold it can be considered necessary for the outcome to occur. None of the conditions met this threshold, although DSGN scored relatively high: 0.835351. I further analysed the condition by looking at the scores across the cases and plotting them against the outcome (innovative solutions) score. Most of the cases scoring low on DSGN did not produce innovative solutions (scoring below 0.5 on INNV) in most cases. The DSGN condition seems to be important, meaning the presence of procedural and institutional rules/arrangements that are designed and applied for co-development of joint outputs seems important for achieving innovative solutions.

**Table 4:** Analysis of Necessity for the Outcome variable INNV (innovative solutions)

Conditions tested	Consistency	Coverage
Collaborative process (PRCS)	0.750605	0.775970
Leadership (LEAD)	0.801453	0.753986
Knowledge sharing (KNWL)	0.761501	0.748810
Institutional design (DSGN)	0.835351	0.870113
Absence of collaborative process (~PRCS)	0.584746	0.602996
Absence of connective leadership (~LEAD)	0.473366	0.541551
Absence of Knowledge sharing (~KNWL)	0.556901	0.605263
Absence of Institutional design (~DSGN)	0.472155	0.483271

The truth table then was constructed within the software (Table 5). Although looking much like a data matrix, truth tables display a different type of information. Rather than denoting a different case to each row, in the truth table each row instead illustrates one of the logically possible combinations of conditions and are being assigned to empirical cases (Schneider and Wagemann 2012). In fsQCA, cases often have partial membership in all rows but they can have a membership of higher than 0.5 in only one row. Cases are thus assigned to this one row to which they fit best (Schneider and Wagemann 2012). For the truth table, the consistency threshold of 0.85 was selected, which is above the recommended level of 0.8 (Ragin 2008; Schneider and Wagemann 2012). Given the limited number of cases, I used a frequency threshold of 1. Initially, 9 configurations (rows from 1 to 9) were obtained for the analysis (Table 5). However, a closer look at these configurations revealed one configuration (row 6) with a case that did not reach the outcome (INNV = 0). This row was excluded from the minimization process.

**Table 5:** Truth table

Rows	LEAD	KNWL	DSGN	PRCS	Number of cases	INNV	Cases	Raw consist.	PRI consist.	SYM consist
1	1	1	1	0	1	1	Case08	0.953271	0.890511	0.890511
2	0	1	1	0	1	1	Case10	0.939759	0.797298	0.797297
3	1	0	1	1	2	1	Case11, Case15	0.930723	0.785047	0.903226
4	1	1	1	1	4	1	Case02, Case07, Case09, Case16	0.895277	0.738462	0.941177
5	0	0	0	1	1	1	Case12	0.89313	0.282051	0.323529
6	1	1	0	1	1	1	Case14	0.869301	0.295082	0.295082
7	1	1	0	0	2	0	Case01, Case04	0.833333	0.294118	0.294118
8	0	1	0	0	1	0	Case06	0.780564	0.113924	0.113924
9	0	0	0	0	3	0	Case03, Case05, Case13	0.546	0.0381355	0.0424527

The remaining configurations then were minimized into solution formula following the application of Boolean minimization. This procedure performed by the fsQCA software results in complex, parsimonious and intermediate solutions illustrating which configurations or pathways lead to innovative solutions. In this analysis, I considered the intermediate solution generated by the software. Intermediate solutions are preferable as they seize a balance between parsimony and complexity while supplementing the empirical information at hand with theory-guided assumptions (Fiss 2011; Schneider and Wagemann 2012). The following intermediate solution with 3 configurations was then derived:

$$\begin{array}{l}{{{\mathrm{KNWL}}}} \ast {{{\mathrm{DSGN}}}} \ast \sim {{{\mathrm{PRCS}}}} + {{{\mathrm{LEAD}}}} \ast {{{\mathrm{DSGN}}}} \ast {{{\mathrm{PRCS}}}} \\ \quad +\, {{{\mathrm{PRCS}}}} \ast \sim {{{\mathrm{LEAD}}}} \ast \sim {{{\mathrm{KNWL}}}} \ast \sim {{{\mathrm{DSGN}}}} \to {{{\mathrm{INNV}}}}\end{array}$$

In Boolean algebra, the * sign indicates multiplication or AND, the + sign indicates OR, the ~ suggests that the condition is absent and illustrates if-then relation (Schneider and Wagemann 2012). These 3 configurations can be interpreted as follows:

### Configuration 1

In a context of limited or unproductive collaborative process, the presence of functional institutional design combined with considerable knowledge sharing leads to innovative solutions.

### Configuration 2

The presence of effective collaborative leadership, functional institutional design and productive collaborative process leads to innovative solutions.

### Configuration 3 (exception)

In a context when collaborative leadership is ineffective, considerable knowledge sharing is not observed and institutional design is not sufficiently functional innovative solutions are possible if the collaborative process is productive.

Although the above interpretation is fairly informative, the current best practice to represent findings from sufficiency analyses in management studies is to use the “configuration chart” (“Fiss charts”) notation system introduced by Ragin and Fiss (2008) and further developed by Fiss (2011). The chart facilitates the comparison across configurations and makes it easier to determine the role of individual conditions (Rubinson 2019). Moreover, the chart allows to illustrate the distinction between core and contributory conditions. The core conditions are represented by “⚫” (presence) and “⊗” (absence); whereas contributing or peripheral conditions are marked with “⚫” (presence) and “⊗” (absence). As already mentioned, the core conditions are those that make up the parsimonious solution indicating conditions that are fundamental to the recipe, cannot be eliminated and must be part of any final solution (Ragin and Sonnett 2005). The smaller glyphs mark a configuration but are not necessary for its delimitation. Finally, blank spaces indicate a “do not care” situation - that is, the condition is not relevant to that configuration. Table 6 visualizes the 3 configurations as well as illustrates the core, peripheral and do not care conditions.

**Table 6:** Solution formula for innovative solutions (consistency threshold 0.8)

	Configurations		
Conditions	C1	C 2	C 3
Collaborative process (PRCS)	⊗	⚫	⚫
Institutional design (DSGN)	⚫	⚫	⊗
Knowledge sharing (KNWL)	⚫		⊗
Leadership (LEAD)		⚫	⊗
Consistency	0.958904	0.865874	0.89313
Raw coverage	0.423729	0.617433	0.283293
Unique coverage	0.0871671	0.288136	0.0738499
Cases	8, 10	7, 16, 15, 2, 9, 11	12
Overall solution consistency		0.863338	
Overall solution coverage		0.7954	

The consistency score for the entire solution term is rather high: 86% (solution consistency 0.863338) of the empirical evidence is in line with the solution term. Furthermore, 79% (solution coverage 0.7954) of the outcome “innovative solutions” is covered by one or more of the three configurations. Consistency and raw coverage measures of each single configuration are also informative here. Raw consistency refers to the proportion of empirical data consistent with the expected outcome, while raw coverage measures the proportion of instances of the outcome that exhibits a certain causal combination or path (Fiss 2011). A solution or path is informative when its consistency is above 0.75–0.80, and its raw coverage is higher than 0.25 (Urueña and Hidalgo 2016): all three configurations exhibit a consistency score way above 0.80, and raw coverage above 0.25.

Overall, the solution shows that institutional design and collaborative process are core conditions in the terms of QCA, pointing out their importance for the outcome. Specifically, functional institutional design is a critical condition for generating innovative solutions in the studies cases. Since the third configuration is rather an exception, institutional design is the only condition that is present in both first and second configurations (Table 6). As also seen with the necessity analysis, institutional design being to some extent a necessary condition, is not sufficient alone for generating innovative solution. In the majority of studied cases that have generated innovative solutions, institutional design is complemented either by knowledge sharing or productive collaborative process and collaborative leadership. Additionally, also the analysis of the absence of innovative solutions albeit rather low consistency and coverage scores, shows the pivotal role of institutional design condition (Table 8). Almost all cases that did not produce innovative solutions, scored also low on institutional design. These cases did not have or did not use sufficiently the basic procedural and institutional rules and arrangement for co-development of innovative solutions.

The first configuration (C1) applies to those cases that did not have regular opportunities to identify, deliberate and make agreements over objectives and shared meanings about innovative solutions (PRCS = 0). While collaborative leadership could be present or absent, what was consequential for these two cases to generate innovative solutions was the presence of functional institutional design complemented with considerable knowledge sharing. The Case 8 - the congestion charge zone (Area C) implemented by the Municipality of Milan in Italy for sustainable mobility (decreased road traffic, improved public transport network, reduced noise and air pollution etc.) had specific institutional arrangements designed to share, mobilize or generate knowledge in support of Area C innovative implementation (Trivellato et al. 2019). So called knowledge orchestrators, internal expert teams and cross-departmental working groups were engaged with each other and international specialists for that purpose. On the other hand, the Case 10, which relates to collaborative efforts of preserving the unique Lake Tahoe in North America, was characterized with problematic collaborative process, conflicts and divisive votes (Imperial 2005). And yet, in view of specific institutional arrangements combined with structured rules for collaboration (federal-interstate compacts, regional plans, environmental threshold carrying capacities) and considerable time invested by collaborating parties in knowledge sharing, innovative solutions were possible in this case.

The second configuration (C2), which explains the majority of successful cases in terms of producing innovative solutions in existing policies, programs and/or practices suggests that the presence of functional institutional design coupled with productive collaborative process and effective collaborative leadership is sufficient to result in innovative solutions. For example one of the cases- the case 16 (Grebbedijk dike reinforcement project in the Netherlands), uniquely covered by this configuration, made use of specific procedural and institutional arrangements to boost innovation. During number of collaborative sessions led by “studio designers”, research-based design methodology was employed to integrate different perspectives on innovative solutions. The discussions and deliberations were informed by the results of in-depth studies on, for example technical innovations, conducted by experts from different sectors. Importantly, the collaborative process was productive to the extent it offered opportunities to deliberate these studies and suggested solutions. The leadership provided by the project manager was instrumental in this process as his skillset of connecting parties, mediating conflicts and steering the project towards integrative and innovative final solution was decisive (Avoyan 2021).

Finally, the third configuration covers only one case and may rather be considered as an exception. The Case 12 (The Tillamook Bay National Estuary Program) is one of the many watershed management programs initiated in the United States to develop and implement Comprehensive Conservation and Management Plans (Imperial 2005). Six watershed management programs are included in this analyses (Cases 10, 11, 12, 13, 14, 15). These collaborative programs differ in their ecological settings, environmental problems, institutional environments, and situational histories, factors found to influence the implementation of watershed management programs etc. (Imperial 2005). Not surprisingly, some of these programs have managed to generate innovative solutions and some did not. If the case 12 would not be the only case that generated innovative solutions while lacking leadership, knowledge sharing and institutional design, further in depth case investigation might have reveal the underlying reasons of such configuration potentially contradicting first and second configurations. However, in this situation, a closer look at the data matrix (Table 7), shows that the scores of absent conditions (LEAD, DSGN, KNWL) are just below the crossover point of falling in the set (conditions = 1). The reason of not reaching the crossover point might be hidden in the limitation of dataset discussed below in the conclusion section.

## Discussion and Conclusions

This study provides insights into the combined effects of four collaborative governance conditions on innovation. I have started the exploration of the likelihood of generating innovative solutions for sustainable environmental management by proposing that collaborative governance might be an useful governance mode to work with. It provides a favourable context for dealing with the challenges of having both a diversity of actors and a common ground for managing differences and developing and implementing new and promising solutions. More specifically, I proposed that four conditions of collaborative governance, namely productive collaborative process, institutional design fit for co-development of innovation, proper knowledge sharing among collaborating parties and leadership characterised with management style of engaging and connecting actors are consequential for innovating. Research findings highlight that for collaborative endeavours to succeed in coming up with innovative solutions, institutional design and collaborative process are critical conditions. Based on the analysis of 16 environmental cases from around the world, on the local, regional, national and supranational level, I find that it is not really necessary to have all the four collaborative conditions in their greatest extent for generating innovative solutions. The functional institutional design coupled with either considerable knowledge sharing or productive collaborative process and effective collaborative leadership may be sufficient to reach the outcome: innovative solutions. Interestingly, in the absence of intensive collaborative process when participants have regular opportunities to deliberate and make agreements over specific issues, the presence of a clear institutional design and structure for following basic rules of co-development and knowledge sharing is pivotal to the production of innovative solutions. On the other hand, specific instances of sharing and managing the knowledge of various collaborating parties may not matter, if the collaborative process is productive enough to provide sufficient opportunities for identification of necessary information, deliberation and consensus.

The above considerations lead to certain implications and conclusions about what matters in collaborative settings when developing innovative solutions for environmental management. First of all, the results of this study illustrate that individual conditions of collaborative governance, such as collaborative process, institutional design, knowledge sharing and collaborative leadership are neither necessary nor sufficient conditions for generating innovative solutions. Only combined in different configurations they may be sufficient for innovative solutions to happen. Second, the results of this study partly confirm what Torfing et al. 2020 concluded in their resent study: institutional design coupled with specific leadership roles is an important condition for realizing collaborative innovation outcomes. This study as well demonstrates the important role of functional institutional design along with productive collaborative process in generating innovative solutions. Third, the configurations discussed in this study clearly illustrate the interconnectedness of collaborative conditions for producing collaborative outputs, such as innovative solutions. For example, knowledge sharing efforts may risk to turn into simple information exchange or absence of results without functioning institutional design mechanisms as shown in the first configuration. Finally, the results of this study provide a guidance to policymakers and practitioners about appropriateness of collaborative governance for generating innovative solutions and suggests conditions that deserve attention.

Although this study deepens the understanding of joint effects of collaborative conditions on innovative solutions in the domain of environment and climate, it does not come without limitations. There are at least three limitations to this study that must be report. First two limitations concern the Collaborative Governance Case databank used to select the cases for analysis, Although the selected cases cover the domain of Environment & climate in different contexts and countries, and have a clear aim of producing innovative solutions, they still do not represent a considerable number of collaborative arrangements globally or within these countries to make the generalization of the findings possible. Therefore, this study provides first insights into this relatively little studied field of research: the link between collaborative governance and innovation in the domain of Environment & climate. In addition, the data of dataset is reported by different researchers without an assessment of intercoder reliability (Ulibarri et al. 2020). This questions the extent of bias related to the data on how reliably collaborative processes are reported. Third limitation concerns the concept of innovative solutions and its calibration for performing QCA. I have used somewhat general definition (also conditioned by the survey question I have used to operationalize the concept of innovative solutions) by encompassing innovative solutions in existing policies, programs and practices for sustainable environmental management. Therefore it is not possible to draw conclusions whether and how the different configurations of collaborative conditions lead to innovation in practices, programs or policies separately. Both for practice and research it would be valuable to investigate whether the found configurations favor more one of these three segments where innovative solutions can be generated. Future research may inquire into individual recipients, being practice or policy, of innovative solutions to shed light on how collaborative conditions contribute to innovative solutions in different segments of environmental management. Future research could take also a wider array of conditions, both characterizing processes within collaborative governance and beyond, into account to test their individual or joint contribution into innovation. Finally, in depth case studies could deepen the knowledge about the configurations found in this study, while the interpretation of configurations outlined above could guide the cases studies as propositions.

## Appendix

Table 7, Table 8

**Table 7:** Data matrix (standardised)

Case number	Innovative solutions	Collaborative process	Collaborative leadership	Knowledge sharing	Institutional design
1	0.8	0.7	0.8	0.8	0.6
2	0.8	1	1	0.8	0.8
3	0.6	0.3	0.4	0.2	0.4
4	0.6	0.7	0.8	1	0.6
5	0.2	0.33	0.6	0.4	0.6
6	0.6	0.45	0.3	0.8	0.6
7	0.8	0.85	1	1	1
8	1	0.6	0.9	1	1
9	0.8	0.9	0.9	1	0.8
10	1	0.7	0.6	0.8	0.9
11	0.8	0.8	0.9	0.6	0.8
12	0.8	0.75	0.7	0.6	0.7
13	0.6	0.55	0.3	0.4	0.6
14	0.6	0.75	0.8	0.8	0.7
15	1	0.8	1	0.6	1
16	1	0.85	1	1	0.9

**Table 8:** Solution for the absence of innovative solutions

Configurations of CGCD		
Conditions	C1	C2
Collaborative process (PRCS)	⊗	⊗
Institutional design (DSGN)	⊗	⊗
Knowledge sharing (KNWL)		⚫
Leadership (LEAD)	⊗	⚫
Consistency	0.514431	0.77971
Raw coverage	0.366828	0.325666
Unique coverage	0.0653754	0.0242131
Cases	3, 6, 13, 5	4, 6, 1
Overall solution consistency	0.525203	
Overall solution coverage	0.391041	
